# Isoliquiritigenin attenuates lipopolysaccharide-induced cognitive impairment through antioxidant and anti-inflammatory activity

**DOI:** 10.1186/s12868-019-0520-x

**Published:** 2019-08-06

**Authors:** Xiaobo Zhu, Jiankun Liu, Shaojie Chen, Jiang Xue, Shanying Huang, Yibiao Wang, Ou Chen

**Affiliations:** 1 Department of Pediatrics, the Second Hospital of Shandong University, #247 Beiyuan Street, Jinan, 250033 People’s Republic of China; 2 Department of Ophthalmology, the Second People’s Hospital of Jinan City, Jinan, 250000 China; 3 The Key Laboratory of Cardiovascular Remodeling and Function Research, Chinese Ministry of Education and Chinese Ministry of Public Health, Shandong University, Jinan, 250012 China; 4 Nursing School, Shandong University, Jinan, 250012 China

**Keywords:** Isoliquiritigenin, Neuroprotection, Oxidative stress, Neuroinflammation, Cognitive impairment, Lipopolysaccharide

## Abstract

**Background:**

Oxidative stress and neuroinflammation are central pathogenic mechanisms common to many neurological diseases. Isoliquiritigenin (ISL) is a flavonoid in licorice with multiple pharmacological properties, including anti-inflammatory activity, and has demonstrated protective efficacy against acute neural injury. However, potential actions against cognitive impairments have not been examined extensively. We established a rat model of cognitive impairment by intracerebroventricular injection of lipopolysaccharide (LPS), and examined the effects of ISL pretreatment on cognitive function, hippocampal injury, and hippocampal expression of various synaptic proteins, antioxidant enzymes, pro-inflammatory cytokines, and signaling factors controlling anti-oxidant and pro-inflammatory responses.

**Results:**

Rats receiving LPS alone demonstrated spatial learning deficits in the Morris water maze test as evidenced by longer average escape latency, fewer platform crossings, and shorter average time in the target quadrant than untreated controls. ISL pretreatment reversed these deficits as well as LPS-induced decreases in the hippocampal expression levels of synaptophysin, postsynaptic density-95, brain-derived neurotrophic factor, superoxide dismutase, glutathione peroxidase, and BCL-2. ISL pretreatment also reversed LPS-induced increases in TUNEL-positive (apoptotic) cells, BAX/BCL-2 ratio, and expression levels of tumor necrosis factor-α, interleukin (IL)-1*β*, IL-6, and C-C motif chemokine ligand 3. Pretreatment with ISL increased the expression levels of phosphorylated (p)-GSK-3*β*, nuclear NRF2, HO-1 mRNA, and NQO1 mRNA, and reversed LPS-induced nuclear translocation of nuclear factor (NF)-*κ*B.

**Conclusions:**

ISL protects against LPS-induced cognitive impairment and neuronal injury by promoting or maintaining antioxidant capacity and suppressing neuroinflammation, likely through phosphorylation-dependent inactivation of GSK-3*β*, enhanced expression of NRF2-responsive antioxidant genes, and suppression of NF-*κ*B-responsive pro-inflammatory genes.

## Background

Cognitive impairment is a common feature of both systemic and neurological diseases, such as sepsis, traumatic brain injury, Alzheimer’s disease (AD), and Parkinson’s disease [1–3]. Growing evidence implicates oxidative stress, neuroinflammation, and ensuing neuronal loss in the initiation and progression of cognitive impairments [4, 5]. Moreover, oxidative stress and inflammation act synergistically to promote cognitive dysfunction [6]. Many experimental models have been established for the mechanistic investigation of cognitive impairment, such as intracerebroventricular (i.c.v.) injection of lipopolysaccharide (LPS), an endotoxin of Gram-negative bacteria. Exposure to LPS triggers a hyperactive immune response with excessive production of inflammatory cytokines, resulting in oxidative stress, deterioration of learning and memory, and other forms of cognitive impairments [7, 8] Hence, LPS-injection is a widely studied model for exploring the molecular basis of cognitive impairments induced by neurological diseases and for testing potential therapeutic strategies.

Isoliquiritigenin (ISL) is a flavanone from Glycyrrhiza uralensis (Chinese licorice) that possess antioxidant, anti-inflammatory, antiviral, antidiabetic, and antitumor activities [9, 10]. Recently, ISL administration was shown to attenuate early brain injury after intracerebral hemorrhage (ICH) by regulating multiple signaling pathways [11]. Learning and memory deficits induced by a high-fat diet (HFD) were also reversed by ISL treatment [12]. In vitro, ISL protected against the production of reactive oxygen species (ROS) and inflammatory cytokines induced by chemical toxins [12, 13]. Nuclear factor erythroid 2-related factor 2 (NRF2), the master regulator of redox homeostasis, could be a key effector for the pharmacological activities of ISL [14, 15]. In HepG2 cells, ISL increased the expression of NRF2 and its downstream detoxification genes [15]. The activation of NRF2 by ISL may be dependent on glycogen synthase kinase (GSK)-3*β*, a negative regulator of NRF2 that is overexpressed in the hippocampus of LPS-treated mice [16]. Moreover, inhibition of GSK-3*β* ameliorated cognitive dysfunction associated with oxidative stress in a mouse model of AD [17].

We speculated that ISL may protect against LPS-induced cognitive impairment by suppressing oxidative stress and inflammation in the hippocampus, and that GSK-3*β* and NRF2 signaling pathways participate in the therapeutic effects of ISL. To test these hypotheses, we established a rat model of cognitive impairment induced by i.c.v. injection of LPS, and assessed the neuroprotective efficacy of ISL and the possible underlying mechanisms. These results provide a promising strategy for the treatment of cognitive impairment in diseases involving oxidative stress and neuroinflammation.

## Results

### ISL reverses LPS-induced cognitive impairment

**Fig. 1:**
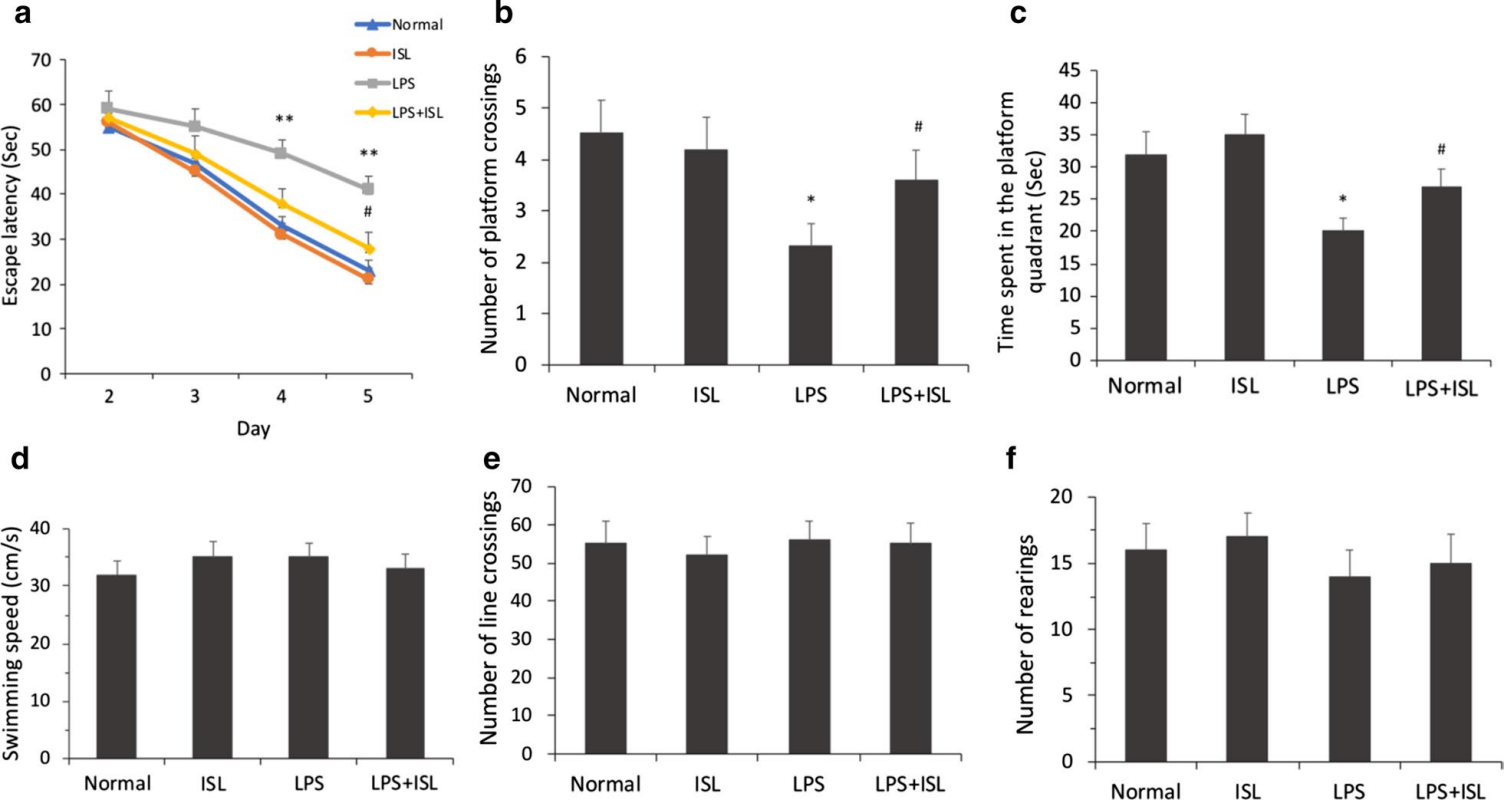
Effects of ISL on cognitive function and spontaneous locomotor activity. Cognitive function was assessed by MWM test. Representative images show **a** the escape latency, **b** the number of platform crossings, **c** the time spent in the platform quadrant and **d** the swimming speed in each group. Spontaneous locomotor activity was evaluated by the open field test. Representative images show **e** the numbers of line crossings and **f** the numbers of rearings in each group. Values are presented as mean ± SEM (*n* = 10). *p < 0.05 and **p < 0.01 vs. normal group, ^#^*p *< 0.05 vs. LPS group

Morris Water Maze (MWM) test was performed to evaluate the effects of ISL pretreatment on LPS-induced cognitive impairment. As shown in Fig. 1a, the escape latency to find the submerged platform improved significantly across the 4-day training session in all groups (*F*_*group* (3, 144)_ = 12.79, *P* < 0.01, *ηp*2 = 0.21; *F*_*day* (3, 144)_ = 69.10, *P* < 0.01, *ηp*2 = 0.59; *F*_*group *×* day* (9, 144)_ = 1.57, *P* = 0.13, *ηp*2 = 0.09). However, escape latency in the LPS group was significantly longer than that in the normal group on days 4 and 5 after LPS injection (both *P *< 0.01). Meanwhile, the LPS + ISL group demonstrated a significantly shorter mean escape latency than the LPS group on day 5 (*P *< 0.05), suggesting that ISL pretreatment partially ameliorated the LPS-induced spatial learning deficit. On day 5 after LPS injection, a probe trial was performed to evaluate spatial memory capacity. Results revealed significant differences among the groups in the number of platform crossings (*F*_(3, 36)_ = 3.24, *P* < 0.05, *ηp*2 = 0.21) and the time spent in the target quadrant (*F*_(3, 36)_ = 5.23, *P* < 0.01, *ηp*2 = 0.30). Rats in the LPS group showed significantly fewer platform crossings (normal group 4.5 ± 0.65 vs. LPS group 2.3 ± 0.45, *P* < 0.05) and a shorter time spent in the target quadrant (normal group 32.0 ± 3.50 s vs. LPS group 20.0 ± 2.09 s, *P* < 0.05) compared to the normal group (Fig. 1b, c), indicating poorer memory for the former platform location. Both the number of platform crossings (vs. LPS + ISL group 3.9 ± 0.43, *P* < 0.05) and target quadrant time (vs. LPS + ISL group 28.3 ± 7.42 s, *P* < 0.05) were greater in the LPS + ISL group compared to the LPS group, suggesting improved spatial memory. Further, these effects could not be explained by motor deficits as there were no significant differences in swimming speed (*F*_(3, 36)_ = 0.37, *P* = 0.80, *ηp*2 = 0.03) among the groups (Fig. 1d). In addition, an open field test performed on day 2 after LPS injection revealed no significant differences in the number of line crossings (*F*_(3, 36)_ = 0.10, *P* = 0.96, *ηp*2 = 0.01; Fig. 1e) and rearings (*F*_(3, 36)_ = 0.42, *P* = 0.74, *ηp*2 = 0.03; Fig. 1f) among the groups.

### ISL reverses LPS-induced synaptic dysfunction

**Fig. 2:**
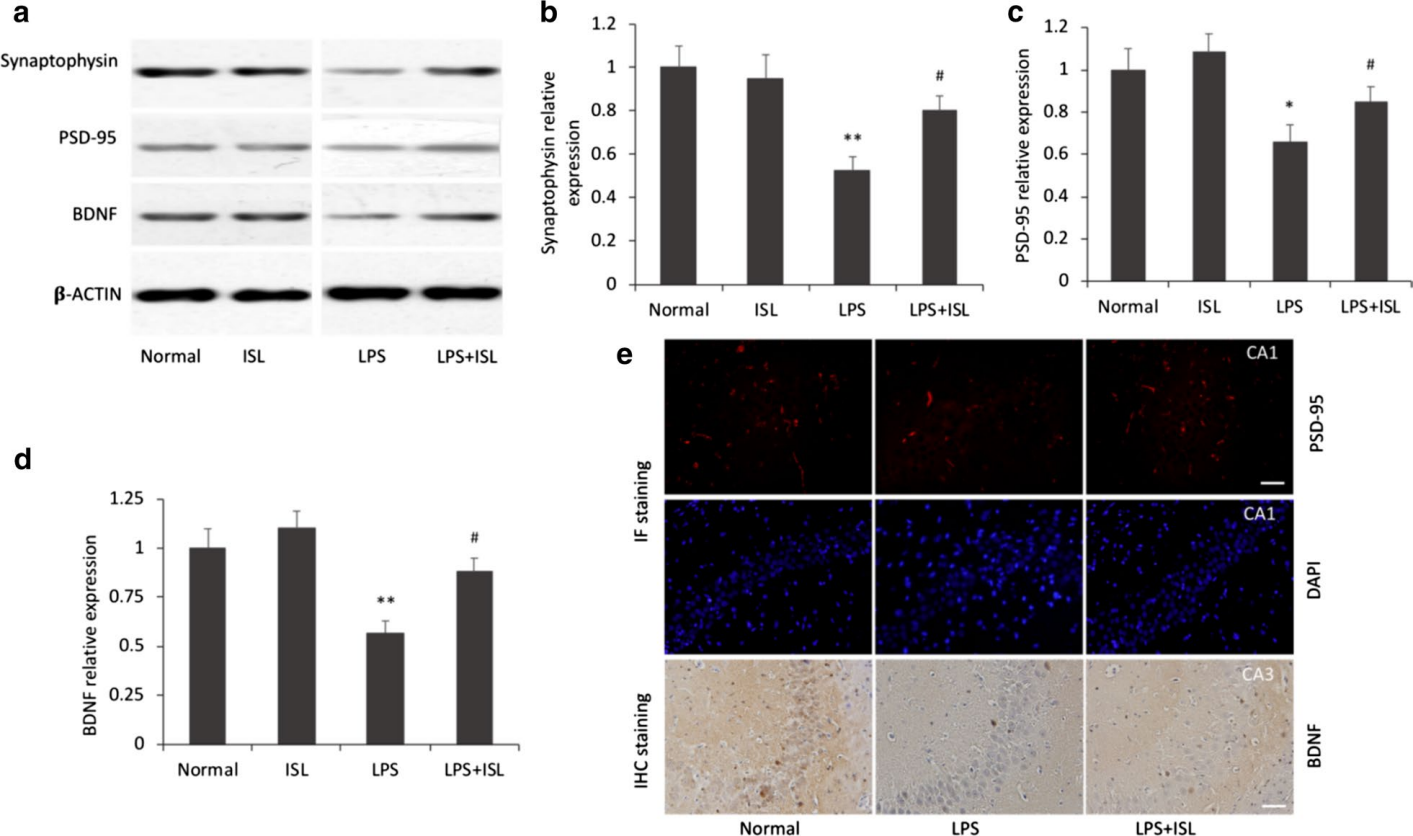
Effects of ISL on LPS-induced synaptic dysfunction. **a**–**d** Western blotting and histograms show the protein levels of synaptophysin, PSD-95 and BDNF in the hippocampus of each group. *β*-Actin was used as a loading control. Values are presented as mean ± SEM (*n* = 6). *p < 0.05 and **p < 0.01 vs. normal group, ^#^*p *< 0.05 vs. LPS group. **e** Representative images show the immunoreactivities of PSD-95 in the CA1 subfield of the hippocampus by IF staining and BDNF in the CA3 subfield by IHC staining. Scale bars = 50 μm

Synaptic dysfunction is a primary feature of cognitive decline. Western blotting assays showed that there were significant differences among the groups in the expression of synaptophysin (*F*_(3, 20)_ = 6.23, *P* < 0.01, *ηp*2 = 0.48), postsynaptic density (PSD)-95 (*F*_(3, 20)_ = 5.53, *P* < 0.01, *ηp*2 = 0.45) and brain-derived neurotrophic factor (BDNF) (*F*_(3, 20)_ = 8.41, *P* < 0.01, *ηp*2 = 0.56) in the hippocampus (Fig. 2a–d). Expression levels of synaptophysin, PSD-95, and BDNF were significantly reduced in the LPS group compared to the normal group (synaptophysin *P *< 0.01; PSD-95 *P *< 0.05; BDNF *P *< 0.01), and ISL pretreatment significantly reversed these LPS-induced decreases (all *P *< 0.05). Alternatively, no significant differences were observed in the levels of synaptophysin, PSD-95, and BDNF between normal and ISL-only groups (all *P* > 0.05).

Immunohistochemical staining was also used to assess the protective effects of ISL on PSD-95 and BDNF expression. As shown in Fig. 2e, the immunoreactivities of PSD-95 and BDNF were significantly lower in the hippocampus of LPS group rats compared to the normal group. Consistent with Western blotting results, ISL pretreatment reversed these decreases in PSD-95 and BDNF immunoexpression. Given the critical importance of PSD-95 and BDNF to synaptic function and plasticity, these results illustrate the protective efficacy of ISL against synaptic dysfunction induced by LPS.

### ISL maintains hippocampal antioxidant capacity under LPS-induced oxidative stress

**Fig. 3:**
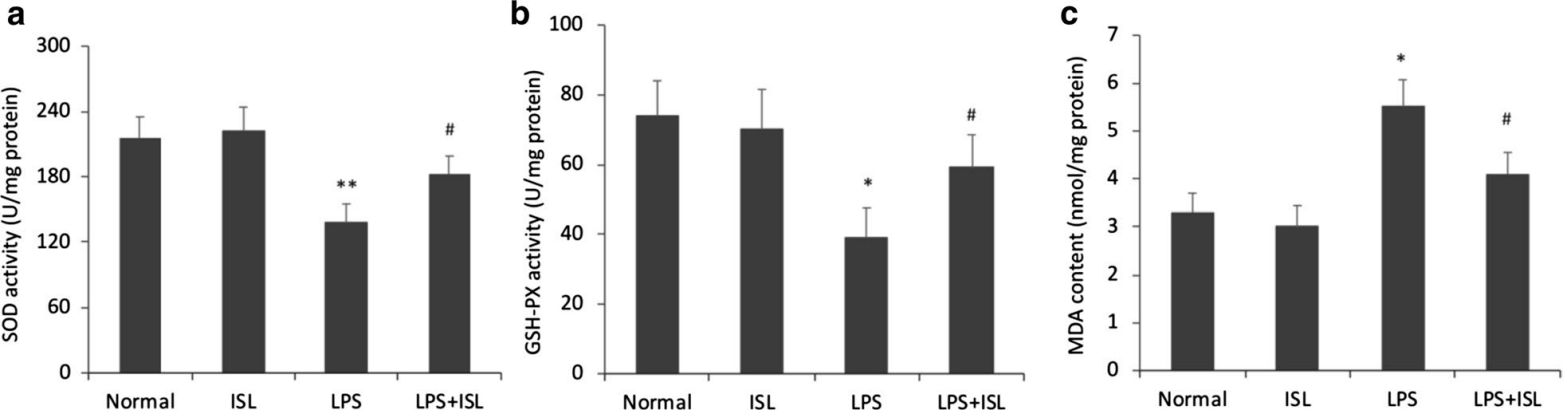
Effects of ISL on hippocampal antioxidant capacity under LPS-induced oxidative stress. Representative images show **a** SOD activity, **b** GSH-PX activity and **c** the content of MDA in each group. Values are presented as mean ± SEM (*n *= 6). *p < 0.05 and **p < 0.01 vs. normal group, ^#^*p *< 0.05 vs. LPS group

We further investigated the effects of ISL on hippocampal antioxidant capacity by measuring superoxide dismutase (SOD) activity, glutathione peroxidase (GSH-PX) activity, and the accumulation of malondialdehyde (MDA), a marker of membrane lipid peroxidation (Fig. 3). There were significant differences among the groups in the activities of SOD (*F*_(3, 20)_ = 4.63, *P* < 0.05, *ηp*2 = 0.41), GSH-PX (*F*_(3, 20)_ = 3.17, *P* < 0.05, *ηp*2 = 0.32) and MDA (*F*_(3, 20)_ = 5.17, *P* < 0.01, *ηp*2 = 0.44). Both SOD (normal 215.0 ± 20.24 vs. LPS 134.2 ± 14.69, *P* < 0.01) and GSH-PX (normal 74.0 ± 10.04 vs. LPS 36.2 ± 7.36, *P* < 0.05) expression levels were significantly reduced by LPS compared to the normal group, while ISL pretreatment significantly reversed these effects (SOD vs. LPS + ISL 189.0 ± 16.30, *P* < 0.05; GSH-PX vs. LPS + ISL 64.8 ± 9.24, *P* < 0.05). There were no significant differences in the levels of SOD (vs. ISL-only 222.1 ± 21.93, *P* > 0.05) and GSH-PX (vs. ISL-only 70.2 ± 11.49, *P* > 0.05) between normal and ISL-only groups. Further, MDA content (normal 3.50 ± 0.40 vs. LPS 5.52 ± 0.57, *P *< 0.05) was significantly elevated in the LPS group compared to controls, an effect abrogated by ISL pretreatment (vs. LPS + ISL 4.07 ± 0.45, *P *< 0.05). No significant differences were observed in the MDA content (vs. ISL-only 3.10 ± 0.43, *P* > 0.05) between normal and ISL-only groups. Thus, ISL appears to sustain hippocampal antioxidant capacity under LPS-induced oxidative stress, possibly accounting for the protective effects against cognitive impairment.

### ISL protects hippocampal neurons against LPS-induced apoptosis

**Fig. 4:**
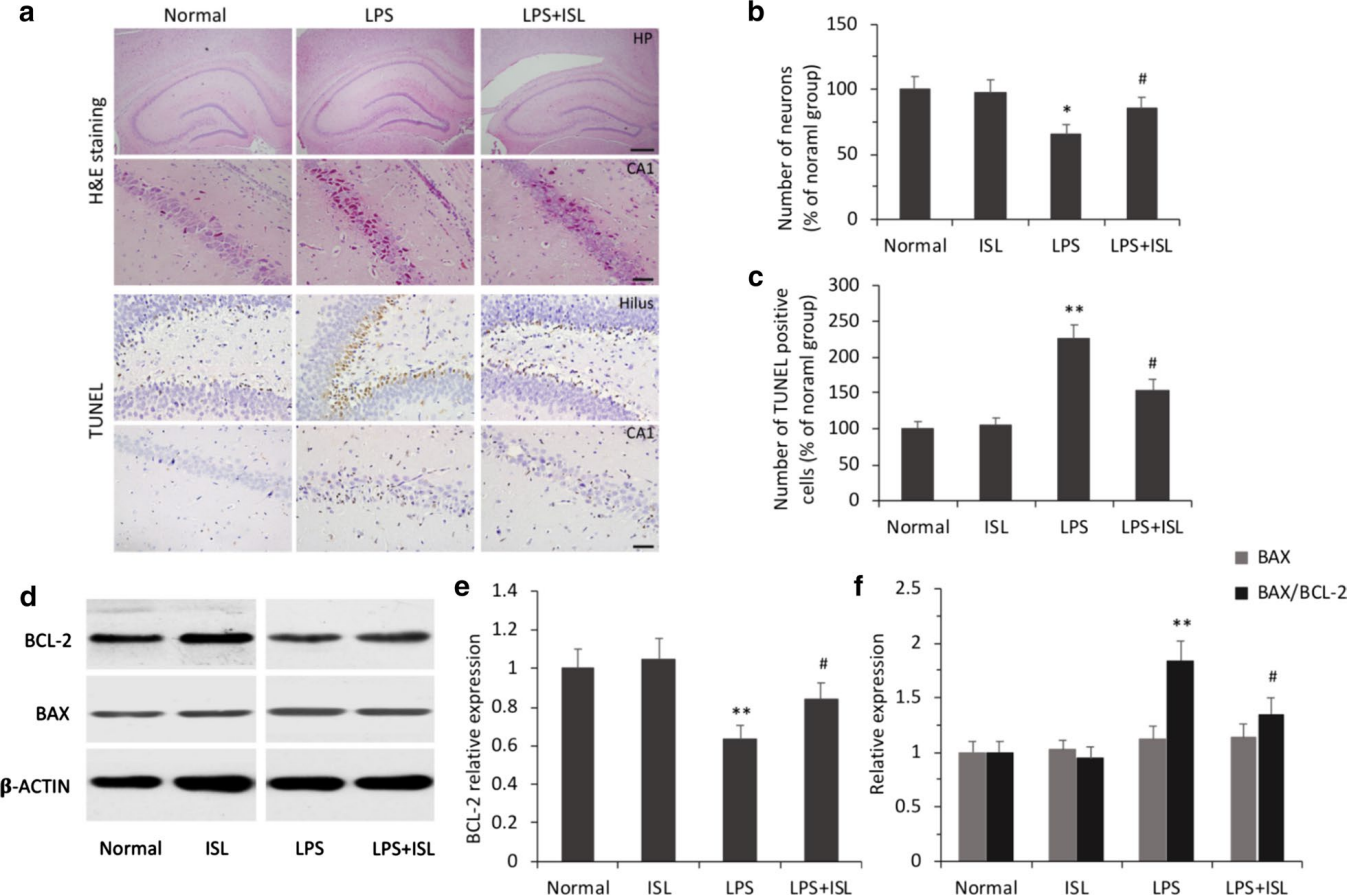
Effects of ISL on LPS-induced neuronal apoptosis. **a** Representative images show neuronal damage in the CA1 subfield and the dentate hilus of hippocampus (HP) using H&E and TUNEL staining. Scale bars = 500 μm (top row), 50 μm (middle and bottom row). **b**, **c** Histograms show the relative neuronal densities and the number of TUNEL-positive cells in the hippocampus. **d**–**f** Western blotting and histograms show the protein levels of BCL-2 and BAX and the ratio of BAX/BCL-2 in the hippocampus of each group. *β*-Actin was used as a loading control. Values are presented as mean ± SEM (*n *= 6). *p < 0.05 and **p < 0.01 vs. normal group, ^#^*p *< 0.05 vs. LPS group

Hematoxylin and eosin (H&E) staining revealed significant differences among the groups in the number of neurons (*F*_(3, 20)_ = 3.34, *P* < 0.05, *ηp*2 = 0.33; Fig. 4a). As expected, neuronal injury was undetectable in the hippocampus of normal rats. Rats in the LPS group exhibited large numbers of neurons with shrunken, fragmented, or punctate shapes. Cell counting analysis revealed that ISL pretreatment significantly attenuated neuronal injury compared to the LPS group (*P *< 0.05; Fig. 4a, b). Further, terminal deoxynucleotidyl transferase- (TdT-) mediated dNTP nick end labeling (TUNEL) assay demonstrated that the number of apoptotic cells differed significantly among the groups (*F*_(3, 20)_ = 14.88, *P* < 0.01, *ηp*2 = 0.69). The number of apoptotic cells was significantly higher in the LPS group than the normal group (*P *< 0.01), and that ISL pretreatment significantly reduced LPS-induced neuronal apoptosis (*P *< 0.05; Fig. 4a, c).

The results of Western blotting showed that there were significant differences among the groups in the expression level of B-cell lymphoma-2 (BCL-2) (*F*_(3, 20)_ = 5.24, *P* < 0.01, *ηp*2 = 0.44) and the ratio of Bcl-2 associated x (BAX) to BCL-2 (*F*_(3, 20)_ = 9.11, *P* < 0.01, *ηp*2 = 0.58; Fig. 4d–f). Consistent with TUNEL staining results, BCL-2 expression was significantly reduced in the LPS group compared to the normal group (*P *< 0.01), an effect reversed by ISL pretreatment (*P *< 0.05; Fig. 4e). Although there were no significant differences in BAX expression among groups (*F*_(3, 20)_ = 0.35, *P* = 0.79, *ηp*2 = 0.049), the BAX/BCL-2 ratio, a widely recognized marker for early apoptosis, was significantly higher in the LPS group than the normal group (*P *< 0.01), whereas ISL pretreatment significantly reduced the BAX/BCL-2 ratio compared to the LPS group (*P *< 0.05; Fig. 4f). Again, there was no significant difference between the normal and ISL-only groups (*P *> 0.05). Thus, ISL protected hippocampal neurons against LPS-induced apoptosis, likely by enhancing or maintaining the expression of various protective factors such as BDNF, BCL-2, SOD, and GSH-PX.

### ISL reduces LPS-induced production of pro-inflammatory cytokines

**Fig. 5:**
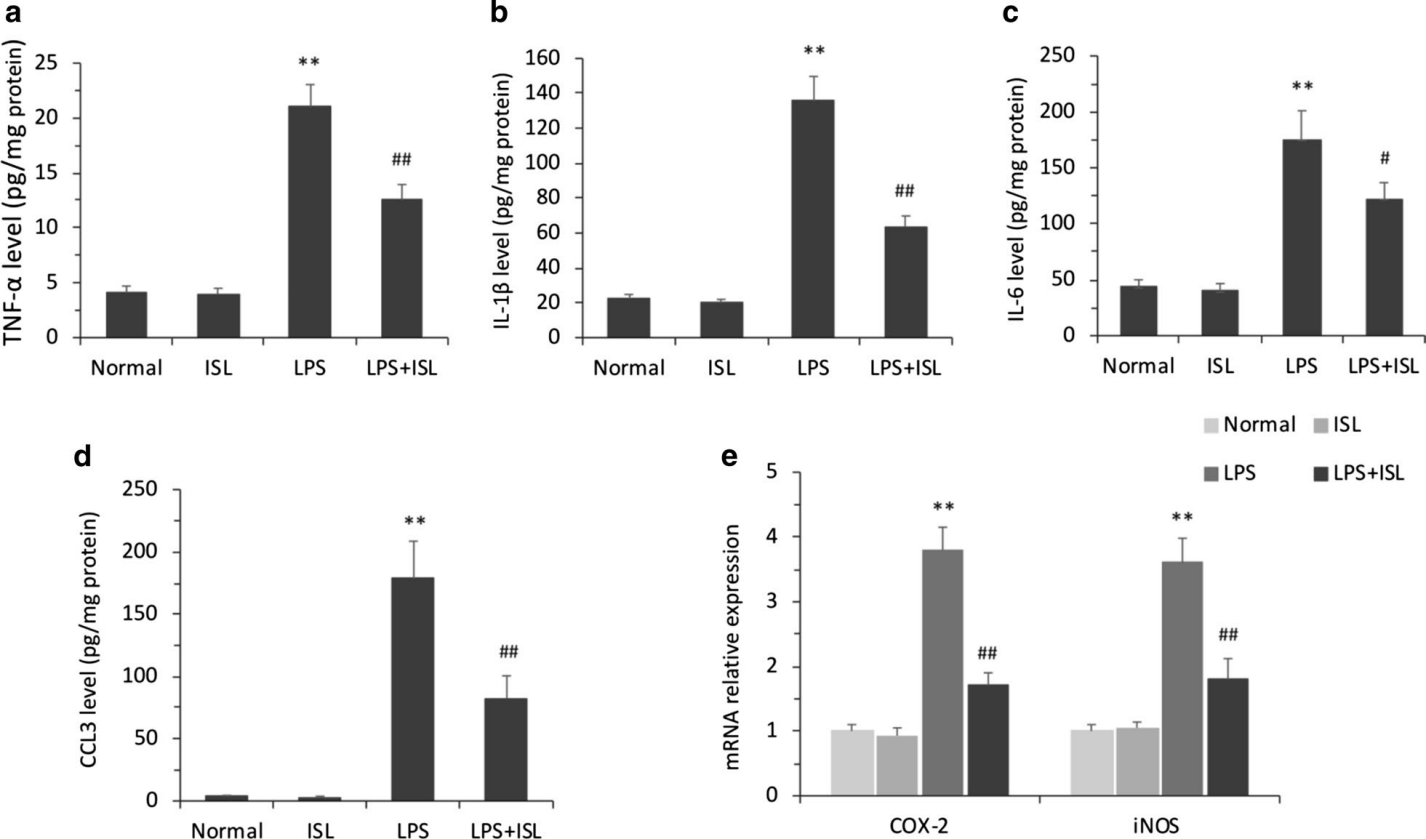
Effects of ISL on the production of pro-inflammatory cytokines induced by LPS. Quantitative analysis by ELISA demonstrates the protein expression level of **a** TNF-*α*, **b** IL-1*β*, **c** IL-6 and **d** CCL3 in the hippocampal homogenates. **e** Quantitative analysis by qRT-PCR demonstrates the mRNA levels of COX-2 and iNOS in the hippocampal homogenates. Values are presented as mean ± SEM (*n *= 6). **p < 0.01 vs. normal group, ^#^*p* < 0.05 and ^##^*p *< 0.01 vs. LPS group

The results of enzyme-linked immunosorbent assay (ELISA) revealed significant differences among the groups in the levels of tumor necrosis factor (TNF)-*α* (*F*_(3, 20)_ = 49.63, *P* < 0.01, *ηp*2 = 0.88), interleukin (IL)-1*β* (*F*_(3, 20)_ = 49.20, *P* < 0.01, *ηp*2 = 0.88), IL-6 (*F*_(3, 20)_ = 39.94, *P* < 0.01, *ηp*2 = 0.86), and C-C motif chemokine ligand 3 (CCL3) (*F*_(3, 20)_ = 44.05, *P* < 0.01, *ηp*2 = 0.87) in the hippocampus (Fig. 5a–d). The expression levels of TNF-*α* (normal 4.05 ± 0.65 vs. LPS 22.47 ± 4.71, *P* < 0.01), IL-1*β* (normal 22.25 ± 2.48 vs. LPS 136.39 ± 13.85, *P* < 0.01), IL-6 (normal 41.50 ± 6.50 vs. LPS 183.40 ± 17.60, *P* < 0.01), and CCL3 (normal 3.52 ± 0.62 vs. LPS 181.37 ± 23.85, *P* < 0.01) were significantly upregulated in the LPS group compared to the normal group, and ISL pretreatment significantly reversed each of these LPS-induced pro-inflammatory responses (TNF-*α* vs. LPS + ISL 12.15 ± 1.32, *P *< 0.01; IL-1*β* vs. LPS + ISL 61.77 ± 6.19, *P *< 0.01; IL-6 vs. LPS + ISL 115.19 ± 8.61, *P* < 0.05; CCL3 vs. LPS + ISL 75.58 ± 7.90, *P *< 0.01). No significant differences were observed between normal and ISL-only groups in the expression levels of TNF-*α* (vs. ISL-only 3.92 ± 0.58, *P *> 0.05), IL-1*β* (vs. ISL-only 19.74 ± 2.15, *P *> 0.05), IL-6 (vs. ISL-only 39.85 ± 6.75, *P *> 0.05), and CCL3 (vs. ISL-only 2.71 ± 0.67, *P *> 0.05).

Further, there were significant differences among the groups in the expression of cyclooxygenase (COX)-2 (*F*_(3, 20)_ = 38.81, *P* < 0.01, *ηp*2 = 0.85) and inducible nitric oxide synthase (iNOS) (*F*_(3, 20)_ = 28.32, *P* < 0.01, *ηp*2 = 0.81) mRNAs. As shown in Fig. 5e, quantitative real time (qRT)-PCR revealed upregulation of COX-2 and iNOS mRNAs by LPS (both *P *< 0.01) and reversal of these increases by ISL pretreatment (both *P *< 0.01).

### ISL pretreatment activates NRF2-dependent transcriptional activity through GSK-3*β* phosphorylation and suppresses nuclear factor (NF)-*κ*B transcriptional activity

**Fig. 6:**
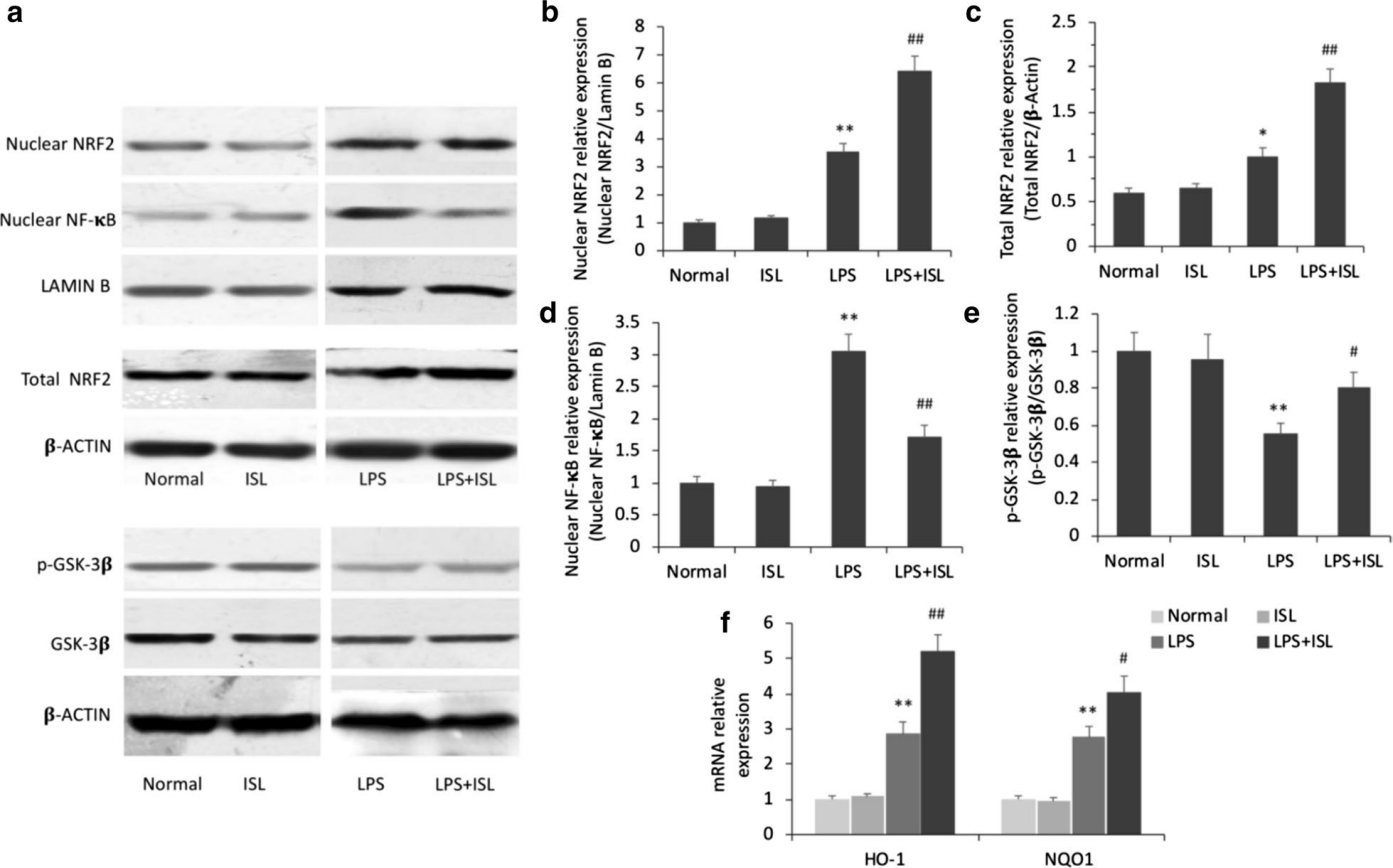
Effects of ISL on GSK-3*β* activity, NRF2 and NF- *κ*B signaling. **a**–**e** Western blotting and histograms show the protein levels of nuclear NRF2, total NRF2, nuclear NF-*κ*B and the ratio of p-GSK-3*β*/GSK-3*β* in the hippocampus of each group. Lamin B and *β*-actin were used as a loading control. **f** Quantitative analysis by qRT-PCR demonstrates the mRNA levels of HO-1 and NQO1 in the hippocampal homogenates. Values are presented as mean ± SEM (*n *= 6). *p < 0.05 and **p < 0.01 vs. normal group, ^#^*p* < 0.05 and ^##^*p *< 0.01 vs. LPS group

Western blotting and qRT-PCR revealed significant differences among the groups in the expression of nuclear NRF2 (*F*_(3, 20)_ = 59.96, *P* < 0.01, *ηp*2 = 0.90), total NRF2 (*F*_(3, 20)_ = 33.33, *P* < 0.01, *ηp*2 = 0.83), nuclear NF-*κ*B (*F*_(3, 20)_ = 35.20, *P* < 0.01, *ηp*2 = 0.84), and phosphorylated (p)-GSK-3*β* (*F*_(3, 20)_ = 6.84, *P* < 0.01, *ηp*2 = 0.51) proteins as well as heme oxygenase (HO)-1 (*F*_(3, 20)_ = 49.96, *P* < 0.01, *ηp*2 = 0.88) and NAD(P)H quinone dehydrogenase 1 (NQO1) (*F*_(3, 20)_ = 42.97, *P* < 0.01, *ηp*2 = 0.87) mRNAs in the hippocampus (Fig. 6). Nuclear immunoexpression of NRF2 was significantly upregulated in the LPS group compared to the normal group (*P *< 0.01), and was further increased by ISL pretreatment (*P *< 0.01; Fig. 6b). The expression level of total NRF2 protein was also enhanced by ISL pretreatment (*P *< 0.01; Fig. 6c). Accordingly, mRNA expression levels of HO-1 and NQO1, cytokines acting downstream of NRF2, were upregulated in LPS + ISL group rats (HO-1 *P *< 0.01; NQO1 *P *< 0.05; Fig. 6f). The nuclear immunoexpression of NF-*κ*B was also significantly upregulated in the LPS group (*P *< 0.01), while ISL pretreatment inhibited nuclear translocation (*P *< 0.01; Fig. 6d). No significant differences were observed in total NRF2, nuclear NRF2, or nuclear NF-*κ*B between normal and ISL-only groups (all *P *> 0.05). Expression of GSK-3*β*, a negative regulator of NRF2, was also examined by Western blotting. The expression of p-GSK-3*β* protein was lower in the LPS group than the normal group (*P *< 0.01), and ISL pretreatment significantly reversed this decrease (*P *< 0.05). Alternatively, ISL pretreatment alone had no effect on the expression of GSK-3*β* (*P *> 0.05; Fig. 6e). Collectively, these results suggest that ISL suppresses the transcriptional activity of NF-*κ*B, thereby reducing pro-inflammatory cytokine production, and inhibits GSK-3*β* activity through phosphorylation, thereby disinhibiting NRF2 and facilitating the expression of NRF2-controlled anti-oxidant genes.

## Discussion

The present study demonstrates that ISL pretreatment ameliorates cognitive impairments caused by neuroinflammation in LPS-treated rats as evidenced by improved spatial learning and memory in the MWM test. This preservation of cognitive function was associated with maintenance of synaptic function and plasticity as indicated by upregulation of synaptic proteins (synaptophysin and PSD-95) and BDNF, a critical modulator of transmission and plasticity. Further, these protective effects of ISL were associated with suppression of oxidative stress and pro-inflammatory cytokine release, likely mediated by upregulation of NRF2 and inhibition of NF-*κ*B transcriptional activity.

Rats receiving ISL pretreatment prior to LPS injection exhibited shorter average escape latency, a greater number of platform crossings, and long average time spent in the target quadrant of the MWM test. Likewise, a recent study found that ISL improved learning and memory on hippocampus-sensitive tasks in young adult ovariectomized rats [18]. ISL also enhanced cognitive performance in ICH model rats as measured by the modified neurological severity score [11] and in ICR mice fed an HFD by reversing inflammation and insulin resistance [12]. Paralleling with previous reports [19, 20], our data showed that LPS injection failed to alter the number of line crossings and rearings in the open field test. Similarly, locomotor activity was not affected by ISL treatment. Such findings indicate that cognitive impairments in the MWM test are not associated with differences in locomotor or exploratory activity. The present study skipped the thigmotaxis during the spatial navigation, because pretrained but not naive rats exhibited reduced periphery swimming in the spatial navigation, and male rats performed thigmotaxis less than females do [21, 22]. Spatial memory in the MWM is dependent on hippocampal synaptic plasticity. Injection of LPS significantly attenuated the expression levels of synaptophysin, PSD-95, and BDNF proteins in the hippocampus, which may have reduced the synaptoplastic capacity of hippocampal circuits. Indeed, both the spatial learning deficit and reduced expression levels of these proteins were reversed by ISL pretreatment. To our knowledge, this is the first report showing a beneficial effect of ISL on synaptic proteins, although a previous report found that the ISL isometric precursor liquiritigenin enhanced the recovery of HIV-1 Tat-mediated synaptodendritic injury via the estrogen receptor [23, 24].

Oxidative stress is characterized by an imbalance between the generation and removal ROS [25]. Oxidative stress is a central pathogenic process in the development of cognitive impairments associated with numerous neurological diseases. In accord with previous studies reporting significant alterations in oxidative stress markers following i.c.v injection of LPS [16, 26], we found significant declines in both SOD and GSH-PX expression in the hippocampus 24 h after LPS treatment, and a concomitant rise in MDA, a marker of lipid peroxidation. Further, ISL maintained antioxidant enzyme expression during LPS-induced neuroinflammation. Consistent with these findings, ISL also restored the oxidant–antioxidant balance in rodent models of brain injury induced by cocaine [27], sepsis [28], ICH [11], and middle cerebral artery occlusion [29]. Oxidative stress is a key contributor to neuronal injury and death, and cognitive capacity is reduced by neuronal cell loss. Numerous studies have reported that injection of LPS induces neuronal apoptosis in the hippocampus [16], including the current study. Further, this LPS-induced neuronal apoptosis was blocked by ISL pretreatment as indicated by H&E staining and TUNEL assay, and was associated with reversal of LPS-induced downregulation of BCL-2, a mitochondrial anti-apoptotic factor, and LPS-induced elevation of the BAX/BCL-2 ratio. Similarly, ISL significantly ameliorated cerebral edema and neurological deficits in ICH model mice [11], an effect accompanied by increases in catalase and SOD activities and reductions in ROS and oxidized glutathione, a marker of oxidative stress. In light of these previous studies, we also speculate that the anti-apoptotic efficacy of ISL and reversal of LPS-induced cognitive impairment are due to inhibition of oxidative stress.

Neuroinflammation also results in cognitive impairments, while numerous clinical and experimental studies have demonstrated cognitive improvements by anti-inflammatory treatment [4, 30]. In a recent paper, ISL reduced the number of myeloperoxidase-positive cells in perihematomal brain tissues of an ICH model [11] and blocked elevated glial fibrillary acid protein expression, a marker of reactive gliosis, induced by methamphetamine in the striatum [31]. In the current study, ISL pretreatment significantly attenuated the production of the pro-inflammatory factors TNF-*α*, IL-1*β*, IL-6, and CCL3 as well as mRNAs encoding COX-2 and iNOS. ISL also inhibited the production of TNF-*α*, IL-1*β*, IL-6, and CCL2 in RAW 264.7 macrophages [32, 33]. Oxidative stress triggers the release of inflammatory cytokines, suggesting a synergistic effect of pro-oxidants and pro-inflammatory factors in cognitive impairment. Therefore, the antioxidant properties of ISL may also contribute to its anti-inflammatory properties.

NRF2 is a master regulator of antioxidant defense responses and is widely expressed in the brain. Under normal physiological conditions, it is sequestered in the cytoplasm in an inactive form. However, oxidative stress enables NRF2 translocation to the nucleus, where it binds to the antioxidant response elements (AREs) of genes encoding peroxiredoxin and phase II detoxification enzymes, such as HO-1, NQO1, and SOD [34, 35]. In the current study, nuclear localization of NRF2 was increased in the hippocampus after LPS injection. In addition, total NRF2 protein was increased, a finding not reported previously in response to LPS [36]. ISL pretreatment further increased nuclear NRF2 and mRNA expression of its downstream genes HO-1 and NQO1. Similarly, NRF2 protein expression, nuclear translocation, and both SOD and catalase activities were upregulated by ISL in an ICH mouse model [11]. There is extensive crosstalk between oxidative stress and inflammatory signaling pathways. Recently, a report verified that NRF2 transactivation by ISL treatment not only reduced the production of oxidative stress markers but also inhibited the activation of NF-*κ*B and ensuing production of inflammatory cytokines [37]. Consistent with the results of Zeng et al. [11], ISL pretreatment also suppressed the nuclear translocation of NF-*κ*B and the production of pro-inflammatory mediators. In several in vitro studies, ISL blocked the activation of the NF-*κ*B pathway by LPS, and reduced NF-*κ*B p65 nuclear translocation [38, 39]. Therefore, the protective effects of ISL against cognitive impairment may be mediated by upregulation of NRF2 signaling and suppression of NF-*κ*B signaling.

GSK-3*β* is a multifunctional kinase implicated in several neuropathological diseases. It is highly active in resting cells and usually inhibits downstream pathways. Phosphorylation within the amino-terminal domain of GSK-3*β* (Ser 9) causes inactivation [40]. A recent study verified that GSK-3*β* downregulates expression of NRF2 protein and genes downstream of NRF2/ARE in brain ischemia–reperfusion injury [41], suggesting that GSK-3*β* is a negative regulatory of NRF2 and the associated antioxidant response. In our study, ISL pretreatment significantly enhanced the level of p-GSK-3*β* (Ser 9) in the hippocampus, indicating GSK-3*β* inactivation, and this effect was associated with upregulation of nuclear NRF2. Moreover, it was previously demonstrated that GSK-3*β* inhibition improves cognition in an AD mouse model [17]. Hence, we speculate that suppression of GSK-3*β* and ensuing activation of the NRF2-dependent antioxidant response is a critical mechanism underlying the protective effect of ISL against LPS-induced cognitive impairment.

## Conclusions

The present study demonstrates that ISL pretreatment can ameliorate cognitive impairments induced by i.c.v. injection of LPS in rats. The protective effects of ISL are associated with strong antioxidant and anti-inflammatory properties, upregulation of proteins related to synaptic function and plasticity, and the attenuation of neuronal apoptosis. At the molecular level, these activities appear to be mediated by inactivation of GSK-3*β*, ensuing upregulation of NRF2 transcriptional activity and suppression of NF-*κ*B transcriptional activity. These results suggest that ISL is a promising candidate therapy for cognitive impairment due to neurological diseases involving neuroinflammation and oxidative stress. Nonetheless, considering the confounding factors in the MWM test, further studies would give us better insights into the underlying mechanisms of cognitive impairments assessed through more than one memory test.

## Methods

### Animal models

Male wistar rats (aged 8 weeks, weighing 180–200 g) were purchased from the Experimental Animal Center of Shandong University, and housed in standardized environment condition with freely access to water and food and a 12 h alternating light and dark. Animal model was constructed by i.c.v. injection of LPS as described in our previous report [42]. Briefly, rats were anesthetized with 10% chloral hydrate (400 mg/kg, i.p.) and treated with buprenorphine (0.04 mg/kg, s.c.) for postsurgical analgesia, and mounted on stereotaxic instrument (Northwest Photoelectric Instruments Plant, Xian, China). After the bregma being exposed, rats were i.c.v. injected with LPS (0.2 mg/kg BW, Escherichia coli serotype 055:B5, Sigma, St. Louis, MO, USA) at a concentration of 50 mg/ml through a Hamilton microsyringe. The stereotaxic coordinates were 1.2 mm posterior, 1.6 mm right, and 2.5 mm deep relative to the bregma. All rats were randomly assigned into the subsequent experiments.

### Experimental protocols

All animals were separated into four groups: normal group, ISL-only group, LPS group, and LPS + ISL group. ISL (20 mg/kg, Shanghai Macklin Biochemical Co., Ltd., Shanghai, China) dissolved in dimethyl sulfoxide (DMSO, ZSGB‐BIO, Beijing, China) solution (25 mg/ml) was intraperitoneally administered at 30 min, 12 h and 24 h prior to LPS injection. The same volume of vehicle (DMSO) was also administrated three times in LPS group. The rats of ISL-only group were injected by ISL alone. The dose of ISL was established by the results of preliminary experiments and published reports [11, 29]. All rats, except those in behavioral tests, were anesthetized with 10% chloral hydrate (400 mg/kg, i.p.) and sodium pentobarbital (100 mg/kg, i.p.), and sacrificed at 24 h and 48 h after LPS injection for molecular experiments and histochemical experiments, respectively.

### Tissue collection and preparation

For Western blotting, ELISA and qRT-PCR (*n* = 6 per group for each experiment), rats were anesthetized by i.p. injection of 10% chloral hydrate (400 mg/kg) and sodium pentobarbital (100 mg/kg), brains were rapidly removed from the skull. The hippocampus was quickly dissected on ice and stored at − 80 °C for later analysis. For histochemical experiments (*n* = 6 per group), rats were perfused transcardially with 4% paraformaldehyde and brain tissues were removed and post-fixed overnight at 4 °C. Tissue samples were embedded with optimal cutting temperature compound. Coronal sections were obtained at the bregma level from − 2.5 to − 3.8 mm, cut at a thickness of 20 μm (1‐in‐6 series, 120 μm apart from each other) with a sliding microtome (Leica Instruments, Germany), and stored at − 20 °C.

### MWM test

MWM test (*n* = 10 per group) was performed to evaluate the spatial learning and memory ability as described previously [29], with slight modification. The instrumentation was obtained from Institute of Material Medicine, Chinese Academy of Medical Sciences, China. The test consisted of a place navigation test and a spatial probe test. The test was performed in a circular pool (120 cm diameter and 50 cm deep) filled with water at 25 ± 1 °C. The pool was divided into four equal quadrants, and an escape platform (10 cm diameter and 1.0 cm beneath the surface of the water) was placed at the center of one quadrant. A digital video camera was mounted above the pool, and swimming activity of the rats was recorded via video tracking software. In the place navigation test, rats were subjected to four trials per day for 4 consecutive days at onset of day 2 after LPS injection. Each rat was placed into the water, facing the pool wall, and given 60 s to locate the hidden platform, if the mice failed to find the platform within 60 s, it would be gently guided to the platform and allowed to remain there for 15 s. On the day 5 after LPS injection, the platform was removed for the probe test. Each rat was allowed to look for the platform in the pool for 60 s. The swimming speed, the number of crossing platforms and the time spent in the target quadrant were recorded for further analysis.

### Open field test

To assess the effect of spontaneous activity of rats in the MWM test, the open field test was performed on the day 2 after LPS injection. Each rat was placed into the corner of a plastic box (90 cm × 90 cm × 40 cm) with the 25 equal sectors on the bottom. A video tracking system (Shanghai Xinruan Information Technology Co., Ltd., Shanghai, China) was used to record the locomotion of rat for 5 min. The open field was cleaned with 5% ethyl alcohol and allowed to dry between tests. The number of line crossings (with all four paws placed into a new square) and the number of rearings (with both front paws raised from the floor) were analyzed.

### Western blotting analysis

Hippocampal tissues were homogenized with a tissue homogenizer in cold lysis buffer. Supernatants were collected after centrifugation at 4 °C (15,000*g* × 15 min). Protein concentrations were measured by using a BCA protein assay kit (Beyotime Biotechnology, Jiangsu, China). Proteins (30 μg per lane) were separated in SDS-PAGE, and then transferred to a polyvinylidene fluoride membrane (Millipore, Billerica, MA, USA). After blocked with 5% skim milk in TBST for 2 h, the membranes were incubated with indicated primary antibody overnight at 4 °C. The primary antibodies used were as follows: rabbit anti-synaptophysin (1:1000, Abcam, Cambridge, MA, USA), PSD-95 (1:1000, Abcam), BDNF (1:1000, Abcam), BCL-2 (1:1000, Abcam), BAX (1:2000, Abcam), NRF2 (1:1000, Cell Signaling Technology, Beverly, MA, USA), NF-*κ*B (1:1000, Cell Signaling Technology), p-GSK-3*β* (Ser9) (1:1000, Cell Signaling Technology), GSK-3*β* (1:1000, Cell Signaling Technology) antibodies, and mouse anti-Lamin B (1:1000, Santa Cruz Biotechnology, Santa Cruz, CA, USA) and *β*-Actin (1:5000, Abcam) antibodies. After three washes, the membranes were treated with species-specific peroxidase-conjugated secondary antibodies for 2 h at room temperature. Immunoreactivities were detected by an enhanced chemiluminescence kit (Millipore) and images were analyzed using an image analyzer (Alpha Innotech, San Leandro, CA, USA). Values were normalized to the amount of *β*-actin or Lamin B in the same samples.

### Immunofluorescence (IF), immunohistochemistry (IHC) and histology analysis

IF and IHC were used to detect the immunoreactivities of PSD-95 and BDNF, respectively. Serial slides were rinsed in PBS and blocked in 5% BSA for 1 h, and reacted with rabbit anti- PSD-95 (1:250, Abcam) or BDNF (1:100, Abcam) antibody overnight at 4 °C. For the immunoreactivity of PSD-95, slides were incubated with Alexa 594-conjugated anti-rabbit IgG (1:500, Invitrogen, Carlsbad, CA, USA) for 1 h and observed using a fluorescence microscope (Olympus, Tokyo, Japan). For the immunoreactivity of BDNF, slides were incubated with a biotinylated anti-rabbit IgG (1:500) for 1 h and avidin-conjugated peroxidase complex (1:200, Vector Laboratories, Burlingame, CA, USA) for 30 min. Positive reaction was visualized using diaminobenzidine tablet sets (ZSGB-BIO) and observed under the light microscope (Olympus).

H&E staining and TUNEL assay were used for the examination of neuronal damage. H&E staining was performed following the standard procedure. TUNEL assay were performed using the In Situ Cell Death Kit, POD (Solarbio, Shanghai, China) according to the manufacturer’s protocol. Images were observed under the light microscope (Olympus). Quantitative analysis of cells was performed by a blinded manner as described in our previous report [43]. In brief, six brain sections per animal were used for counting at a 200× magnification. The number of target cells was counted twice on two differently visual fields of each subfield of hippocampus, including the hilus of dentate gyrus, cornu ammonis 3 (CA3) subfield and CA1 subfield. The value was expressed as the ratio of mean outcome in experimental group to that in the normal group.

### Measurements of SOD activity, GSH-PX activity and MDA content

Hippocampal tissues were homogenized in PBS and supernatants were obtained after centrifugation at 4 °C (15,000*g* × 30 min). The activities of SOD and GSH-PX and the content of MDA were examined according to the manufacturer’s instructions of total SOD assay kit (Hydroxylamine method), GSH-PX assay kit (Colorimetric method) and MDA assay kit (TBA method). All kits were purchased from Nanjing Jiancheng Bioengineering Institute, Nanjing, China.

### ELISA

The hippocampal tissues were treated as mentioned above. The levels of TNF-*α*, IL-1*β*, IL-6, and CCL3 were quantified by ELISA kit according to the manufacturer’s instructions (TNF-*α*, IL-1*β* and IL-6 from Invitrogen; CCL3 from Peprotech, Rocky Hill, NJ, USA). Results are described as pictogram per milligram total protein.

### qRT-PCR

Total RNA was extracted from dissected hippocampus using Trizol reagent (Invitrogen). RNA concentration was quantified by measuring the absorbance at 260 nm. First-strand cDNA was generated using the Reverse Transcription System (Qiagen, Valencia, CA, USA). The qPCR was performed using Maxima SYBR Green dye (Fermentas, Glen Burnie, MD, USA) in an Eppendorf thermocycler (Hamburg, Germany). The qPCR reaction mixture comprised 10 μl of Maxima SYBR Green qPCR Master Mix, 2 μl of cDNA, and 0.4 μl of 10 μmol/l forward and reverse primers, made up to a final volume of 20 μl with ultrapure water. The qPCR primers used were as follows: COX-2 forward, GGTTCACCCGAGGACTGGGC, and reverse, CGCAGGTGCTCAGGGACGTG; iNOS forward, CCAACCTGCAGGTCTTCGATG, and reverse, GTCGATGCACAACTGGGTGAAC; HO-1 forward, TGCTCGCATGAACACTCTGGAGAT, and reverse, ATGGCATAAATTCCCACTGCCACG; NQO1 forward, GTGAGAAGAGCCCTGATTGT, and reverse, CCTGTGATGTCGTTTCTGGA; and GAPDH forward, CCCTTCATTGACCTCAACTACA, and reverse, GCCAGTAGACTCCACGACATA. The standard PCR conditions were 95 °C 10 min, followed by 40 cycles of 95 °C 15 s, 60 °C 30 s, and 72 °C 30 s. Each sample was run in triplicate. The expression of the target genes was normalized to GAPDH as an internal control. Gene expression was analyzed using the 2(−Delta Delta ct) method [44].

### Statistical analysis

Statistical analysis was performed using SPSS version 21.0 software (SPSS, Inc., Chicago, IL, USA) and all data were presented as mean ± standard error of mean (SEM). For the escape latency data in MWM test, statistical comparisons were conducted using two-way analysis of variance (ANOVA) (group and trial time) with repeated measures (trial days). All other data were analyzed with one-way ANOVA. Post hoc analyses were performed using Fisher’s least significant difference (LSD) test or Dunnett’s T3 test to compare groups. Differences with *p* < 0.05 were considered significant.

## Data Availability

The datasets and material used during the current study are available from the corresponding author on reasonable request.

## Abbreviations

AD: Alzheimer’s disease
ANOVA: analysis of variance
AREs: antioxidant response elements
Bax: Bcl-2 associated x
BCL-2: B cell lymphoma-2
BDNF: brain-derived neurotrophic factor
CA3: cornu ammonis 3
CCL3: C-C motif chemokine ligand 3
COX: cyclooxygenase
DMSO: dimethyl sulfoxide
ELISA: enzyme-linked immunosorbent assay
GSH-PX: glutathione peroxidase
GSK: glycogen synthase kinase
H&E: hematoxylin and eosin
HFD: high-fat diet
HO: heme oxygenase
i.c.v.: intracerebroventricular
ICH: intracerebral hemorrhage
IF: immunofluorescence
IHC: immunohistochemistry
IL: interleukin
iNOS: inducible nitric oxide synthase
ISL: isoliquiritigenin
LPS: lipopolysaccharide
LSD: least significant difference
MDA: malondialdehyde
MWM: Morris Water Maze
NF: nuclear factor
NQO1: NAD(P)H quinone dehydrogenase 1
NRF2: nuclear factor erythroid 2-related factor 2
p-: phosphorylated
PSD: postsynaptic density
qRT: quantitative real time
ROS: reactive oxygen species
SEM: standard error of mean
SOD: superoxide dismutase
TNF: tumor necrosis factor
TUNEL: terminal deoxynucleotidyl transferase- (TdT-) mediated dNTP nick end labeling
